# Retinal hyperreflective foci in Fabry disease

**DOI:** 10.1186/s13023-019-1267-2

**Published:** 2019-12-26

**Authors:** Yevgeniya Atiskova, Rahman Rassuli, Anja Friederike Koehn, Amir Golsari, Lars Wagenfeld, Marcel du Moulin, Nicole Muschol, Simon Dulz

**Affiliations:** 1 Department of Ophthalmology, University Medical Center Hamburg-Eppendorf, Martinistraße 52, 20246 Hamburg, Germany; 2 Department of Pediatrics, University Medical Center Hamburg-Eppendorf, Hamburg, Germany; 3 Department of Neurology, University Medical Center Hamburg-Eppendorf, Hamburg, Germany

**Keywords:** Fabry disease, SD-OCT, Retina, Hyperreflective foci, Vessel tortuosity

## Abstract

**Background:**

Fabry disease (FD) is an X-linked inherited storage disorder caused by deficiency of lysosomal alpha-Galactosidase A. Here we describe new retinal findings in patients with FD assessed by Spectral domain optical coherence tomography (SD-OCT) and their possible clinical relevance.

**Methods:**

54 eyes of 27 FD patients and 54 eyes of 27 control subjects were included. The ophthalmic examination included visual acuity testing, tonometry, slit lamp and fundus examination. SD-OCT imaging of the macula was performed in all subjects. Central retinal thickness and retinal nerve fiber layer analysis were quantified. Vessel tortuosity was obtained by a subjective scoring and mathematically calculated. Inner retinal hyperreflective foci (HRF) were quantified, clinically graded and correlated with a biomarker of Fabry disease (lyso-Gb3).

**Results:**

In comparison to an age-matched control group, a significant amount of HRF was identified in macular SD-OCT images in FD patients. These HRF were localized within the inner retinal layers. Furthermore, lyso-Gb3 levels correlated significantly with the quantitative evaluation of HRF (*p* < 0,001). In addition, the vessel tortuosity was remarkably increased in FD patients compared to control persons and correlated significantly with lyso-G3 levels (*p* = 0.005). A further subanalysis revealed significantly higher HRF and vessel tortuosity scores in male patients with the classic FD phenotype.

**Conclusions:**

The observational, cross sectional, comparative study describes novel intraretinal findings in patients with FD. We were able to identify suspicious HRF within the inner retinal layers. These findings were not accompanied by functional limitations, as visual acuity remained unchanged. However, HRF correlated well with lyso-Gb3, a degradation product of the accumulating protein Gb3 and might potentially indicate Gb3 accumulation within the highly metabolic and densely vascularized macula.

## Introduction

Fabry disease (FD) is an X-linked multisystemic lysosomal storage disorder characterized by decreased or deficient activity of the enzyme α-Galactosidase A. This results in progressive deposition of sphingolipids within a multitude of cells and organs. The prevalence of FD is estimated between 1/40.000 and 1/117.000 [1–4].

FD patients present with a wide spectrum of phenotypes – from mono-or oligosymptomatic cases to severe multi-organ involvement [5]. Severe, e.g. nonsense mutations typically lead to a classical, severe phenotype, whereas there are special missense mutations that lead to oligosymptomatic forms of the disease [6–12].

Early systemic manifestations of FD include superficial vascular lesions (angiokeratomas), intermittent episodic pain sensation in the extremities (acroparesthesia), proteinuria, hypohidrosis, heat/cold and exercise intolerance as well as gastrointestinal symptoms [13–16]. As the disease progresses, progressive renal dysfunction may result in end-stage renal disease (ESRD) requiring dialysis [17–19]. Cardiac complications include left ventricular hypertrophy, arrhythmias, heart failure and acute myocardial infarction [20–22]. Cerebrovascular occlusive events include transient ischemic attacks or early stroke, leading to premature death [23, 24].

The availability of enzyme replacement therapy (ERT) with agalsidase alfa and beta has effectively reduced mortality and morbidity in patients with FD over the last decades [25–27].

Ocular manifestations occur most superficially as “cornea verticillata” (CV), a vortex keratopathy representing the most commonly reported feature of ophthalmologic disease in FD [28, 29]. Conjunctival vessels often present with unspecific tortuosity and infrequently with aneurysmal dilatations [30]. A small fraction of patients (9.8% of females and 23.1% of males) present with a “spoke-like” lens opacity (Fabry cataract) at the level of the posterior capsule [30]. Patients with FD show retinal vascular tortuosity, which has shown to correlate well with disease severity [31]. Computer assisted analysis of retinal vasculature demonstrated the potential of retinal vessel tortuosity as a noninvasive marker of disease progression [32, 33]. As ocular alterations routinely do not alter visual function, rare retinovascular incidences such as arterial and venous occlusion can lead to profound visual deterioration [34].

Monitoring of organ dysfunction and damage in a lysosomal storage disorder such as FD plays a pivotal role to estimate prognosis and planning of specific and symptomatic therapy. The eye and its vascular status represent an easily accessible end organ, where rapid and noninvasive imaging methodologies can be applied.

The provided study reveals novel insight into retinovascular and intraretinal features of FD, with special emphasis on the herein first described inner retinal hyperreflective foci (HRF), which can be apictured by spectral-domain optical coherence tomography (SD-OCT). Subjective as well as objective methods were applied to describe retinovascular and intraretinal features to implement the latter into clinical practice.

## Methods and material

The study was approved by the medical ethics committee of the Ärztekammer Hamburg, Germany and followed the tenets of the declaration of Helsinki for research involving human subjects. Informed consent was obtained from all participants of the study.

54 eyes of 27 patients with genetically confirmed FD and an age matched control group of 54 eyes of 27 healthy volunteers were included into the prospective observational, cross sectional, comparative study. The control group was recruited among healthy coworkers at the University of Hamburg-Eppendorf. Ocular comorbidities resolved in exclusion from the study. Patients demographics are displayed in Table 1 including age, gender and genotype. Lyso-Gb3, an established serum biomarker in FD that correlates well with disease severity, was obtained from all patients [35]. All patients and the control group underwent a detailed ocular examination including best-corrected visual acuity (BCVA) testing, non-contact tonometry, slit-lamp biomicroscopy (including the assessment of CV, conjunctival tortuosity and lens opacification), and funduscopy. The finding of a CV was further subdivided into four clinical grades according to Orlando et al. [36].

**Table 1:** Demographic and clinical data of all investigated FD patients

Patient	Age [years]	Sex	Mutation	Lyso-Gb3 [ng/ml]	ERT^1^
1	32	f	p.N320I	4.2	no^a^
2	56.9	f	c.718_719delAA	10.0	agalsidase alfa for 123 mo
3	26.7	m	c.718_719delAA	26.6	agalsidase beta for 14 mo^b^
4	27.8	m	p.R227X	19.7	agalsidase alfa for 96 mo
5	23.1	m	p.N215S	2.8	no
6	52.9	f	p.N215S	1.6	no
7	11.9	m	p.D313Y	0.5	no
8	42	f	p.D313Y	0.5	agalsidase alfa for 23 mo
9	40	m	p.Q327L	24.0	agalsidase alfa for 92 mo
10	34	m	c.717_718delAA	85.8	agalsidase alfa for 80 mo
11	57.2	f	c.717_718delAA	n.a.	agalsidase alfa for 72 mo
12	58.6	m	p.E341K	33.9	agalsidase beta for 28 mo
13	26.5	f	p.D313Y	0.6	agalsidase alfa for 30 mo
14	50.7	f	p.D313Y	3.9	agalsidase alfa for 32 mo
15	30.6	f	p.D313Y	0.6	no
16	65.3	m	p.N215S	5.5	agalsidase beta for 12 mo
17	48.7	f	c.718_719delAA	6.3	agalsidase alfa for 25 mo
18	56.4	m	p.I384N	20.6	agalsidase alfa for 120 mo
19	39.3	f	p.D313Y	0.7	agalsidase alfa for 7 mo
20	11.2	m	p.D313Y	0.7	no
21	53	f	c.1277_1278delAA	11.8	agalsidase alfa for 47 mo
22	51.6	m	p.P205T	28.9	agalsidase alfa for 28 mo
23	55.1	m	p.A143T	0.6	no
24	59.9	f	p.Q327L	6.9	agalsidase alfa for 124 mo
25	37.6	m	p.R227Q	36.4	agalsidase alfa for 118 mo
26	33	f	p.E341K	1.6	no
27	44.8	m	p.L89del	107.0	no

at time of ocular examination

*f* female, *m* male, *ERT* Enzyme replacement therapy, *mo* months, *n.a.* not available

^a^(agalsidase alfa for 19 mo until 4 mo before ocular exam)

^b^(switched from agalsidase alfa)

To assess the retinal morphology, SD-OCT imaging (Spectralis OCT, Heidelberg Engineering, Heidelberg, Germany) was performed and acquired images were further processed and analysed with ImageJ (Rasband, W.S., ImageJ, U. S. National Institutes of Health, Bethesda, Maryland, USA).

**Fig. 1:**
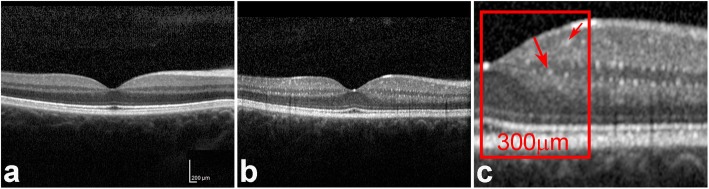
A representative central SD-OCT slice of the right eye of a healthy control person is shown in (**a**). The macular SD-OCT slide of a left eye of FD patient (**b**) shows significant HRF. A magnification of the parafoveal region, highlights numerous HRFs (red arrows) within the inner retinal layers (**c**). Quantification was performed by counting an area of 300 μm temporal (red box) and nasal to the foveal center

Central retinal thickness (CRT) evaluation was attained by analysing macular scans, which were acquired using horizontal raster pattern scans (20 × 20° (5.4 × 5.4) scan field). CRT values were extracted from the central Early Treatment Diabetic Retinopathy Study (ETDRS) subfield. The retinal nerve fiber layer (RNFL) was obtained performing an additional automatized “RNFL scan”. For statistical correlation analysis, averaged CRT and RNFL measurements of both eyes were calculated. Manual quantification of HRF, *referred to as quantitative score of HRF*, was performed by a blinded examiner in a standardized fashion. The temporal and nasal parafoveal region (0-300 μm) were outlined in a foveal cross section with the aid of ImageJ and quantified with the counting tool at 300% magnification (Fig. 1). Inner retinal HRF were defined as hyperreflective foci with similar reflectivity as the retinal pigment epithelium and a diameter > 10 μm. Potential HRF with a shadow were excluded from quantification to ensure exclusion of retinal arterioles and venules. In order to provide clinical feasability, SD-OCT cross-sections of patients with FD as well as an age-correlated control group were graded by the three independent ophthalmologists as absent (0), mild (1), moderate (2), severe (3), referred to as *subjective score of HRF.*

**Fig. 2:**
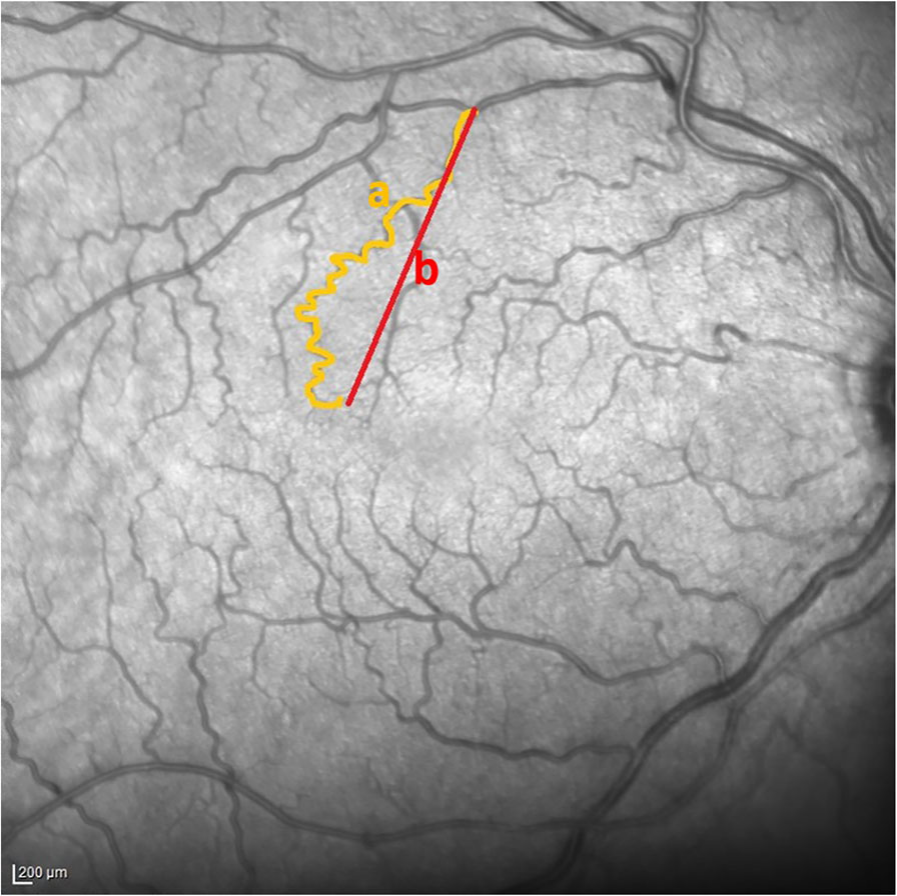
A cSLO image of a patient with FD. Retinal vessel tortuosity was analysed by measuring the true length (**a**) and the end-to-end length (**b**) of the retinal vessels and dividing a/b

Retinal vessel tortuosity was analysed on basis of cSLO images acquired during the SD-OCT imaging process. In each picture the user subjectively chose the three most tortuous vessels. The designated branching retinal vessels were selected to obtain the *calculated vessel tortuosity score* by measuring the true length (a) and the end-to-end length (b) of the retinal vessels and dividing a/b (Fig. 2).

Prior to the calculated analyses, a clinical grading of retinal vessel tortuosity, referred to as *subjective vessel tortuosity score,* was performed by three independent ophthalmologists as absent (0), mild (1), moderate (2), severe (3) on the basis of the acquired cSLO images similar to previous described retinal vessel tortuosity grading [34].

Statistical analysis was performed with SPSS (v15, IBM, New York, NY, USA). The level of significance was *p* < 0.05 in all statistical tests. The Wilcoxon-Mann–Whitney *U* test (nonparametric two-independent sample test) was used to compare all retinal parameters between FD patients and the control group, whereas the Independent T-Test was applied in the normally distributed analyses. Correlation analyses were performed using Spearman Rank Order Test.

## Results

In the present study, we analysed the retinal phenotype of patients with genetically confirmed FD. Demographic data of the 27 patients are shown in Table 1. The median age was 42 years (range: 11.2–65.3 years). The gender ratio was balanced (51.9% male, 48.1% female). The median age of the control group was 39.1 years (range: 11.3–64.3 years). The gender ratio was matched to the Fabry cohort. Up to the time of data acquisition 19 of the 27 FD patients received enzyme replacement therapy. All analysed patients presented with a BCVA of 20/25 or better in both eyes. The anterior segment examination revealed conjunctival vessel tortuosity (various degrees of tortuosity including vascular anomalies e.G. *ampullae*) in 14 eyes of 7 FD patients. One patient presented with a mild subcapsular Fabry cataract. CV was noticed in 19 of 27 patients (mean grade = 1.2; range: 0–4). Of note, CV was mainly found in patients with classical Fabry mutations (15/16), while only 4 of the 11 patients with the oligosymptomatic or non-classical genetic variants p.D313Y and p.A143T or the cardiac variant p.N215S presented with CV.

In the macular SD-OCT-images intraretinal HRF were observed within the inner retinal layers (ganglion cell layer, inner plexiform layer, inner nuclear layer, outer plexiform layer) in most analysed FD patients. In SD-OCT images of the retinas of FD patients a significantly higher quantitative score of HRF (mean = 82.0, range: 18.0–221.0) was obtained in the defined parafoveal region compared to the age-matched control group (mean = 9.0; range: 1.0–17.0). Furthermore a significantly higher subjective score of HRF was seen in FD patients (mean = 1.33; range: 0.42–3) compared to the control group (mean = 0.33; range: 0–0.75); (Table 2). 20 of 27 FD patients revealed a clinically remarkable degree of HRF (grade 1–3), whereas none of the control subjects presented with a clinically relevant degree of HRF. Linear regression analysis showed a significant correlation between the subjective score of HRF and the quantitative score (*r* = 0.7; *p* < 0.001). The quantitative score of HRF showed a significant correlation with the laboratory parameter lyso-Gb3, which is used for monitoring of FD in clinical practice (*r* = 0.7; *p* < 0.001). Furthermore, a significantly higher number of HRF was found in male FD patients compared to females (Table 3). The mean quantitative score of HRF amounted 117.2 (range: 36.5–221.0) in males and 64.69 (range: 18.0–122.0) in females (*p* = 0.011). In addition, the subjective score of HRF was significantly higher in males (mean = 2.62; range: 0.42–3) than females (mean = 1.25; range: 0.67–1.75; *p* = 0.049).

**Table 2:** Comparison of the investigated retinal parameters between FD patients and control group

	Patients			Control			*P*-Value
	N	Min/Max	DIST.	N	Min/Max	DIST.	
Central retinal thickness	27	0.00/305.50	276.00 (266.25/284.25)	27	191.50/307.50	275.00 (267.00/283.25)	0.795^†^
Retinal nerve fiber layer thickness	27	0.00/117.00	92.00 (86.50/100.50)	27	76.50/188.50	97.00 (92.50/101.25)	0.139^†^
Objective calculated vessel tortuosity score	27	1.17/2.02	1.34 (1.29/1.48)	27	1.14/1.38	1.19 (1.18/1.21)	<0.001^†^
Subjective obtained vessel tortuosity score	27	0.33/3.00	1.42 (0.92/2.58)	27	0.00/1.33	0.17 (0.00/0.38)	<0.001^†^
Quantitative score of hyperreflective intraretinal deposits	27	18.00/221.00	82.00 (49.75/116.25)	27	1.00/17.00	9.00 (5.25/13.00)	<0.001^†^
Subjective obtained score of hyperreflective intraretinal deposits	27	0.42/3.00	1.33 (0.96/2.62)	27	0.00/0.75	0.33 (0.12/0.46)	<0.001^†^

^*^Normally distributed. Mean ±SD is displayed under DIST. Independent T-Test is used

^†^Not normally distributed. Median (Q25/Q75) is displayed under DIST. Mann-Whitney Test is used

**Table 3:** Comparison of the investigated retinal parameters between female and male FD patients

	Female			Male			*P*-Value
	N	Min/Max	DIST.	N	Min/Max	DIST.	
Central retinal thickness	13	241.50/287.50	267.50 (257.50/274.50)	14	0.00/305.50	283.25 (276.62/289.62)	0.007^†^
Retinal nerve fiber layer thickness	13	0.00/99.50	90.50 (87.00/93.00)	14	0.00/117.00	99.25 (86.38/103.62)	0.126^†^
Objective calculated vessel tortuosity score	13	1.17/1.46	1.31 ± 0.08	14	1.20/2.02	1.49 ± 0.22	0.013^*^
Subjective obtained vessel tortuosity score	13	0.33/2.25	1.08 (0.67/1.42)	14	0.58/3.00	2.58 (1.42/3.00)	0.005^†^
Quantitative score of hyperreflective intraretinal deposits	13	18.00/122.00	64.69 ± 31.41	14	36.50/221.00	117.18 ± 62.12	0.011^*^
Subjective obtained score of hyperreflective intraretinal deposits	13	0.67/1.75	1.25 (0.92/1.50)	14	0.42/3.00	2.62 (1.12/2.92)	0.049^†^

^*^Normally distributed. Mean ±SD is displayed under DIST. Independent T-Test is used

^†^Not normally distributed. Median (Q25/Q75) is displayed under DIST. Mann-Whitney Test is used

When we surveyed the tortuosity of the retinal vessels, a significantly higher calculated vessel tortuosity score was detected in FD patients (mean = 1.34; range: 1.17–2.02) compared to the age-matched control group (mean = 1.19; range: 1.14–1.38; *p* < 0,001). Also the subjective clinical score revealed a more severe vessel tortuosity score in FD patients (median = 1.42; range: 0.33–3.0) than in the control group (median = 0.17; range 0–1.33; *p* < 0.001; Table 2).

Furthermore, a significant positive correlation between the subjective score of retinal vessel tortuosity and the calculated values of the vessel tortuosity was visible (*r* = 0.6; *p* < 0.001).

The calculated vessel tortuosity score (*r* = 0.54; *p* = 0.005) also shows a moderate positive correlation with the laboratory parameter lyso-Gb3.

A significantly higher vessel tortuosity was noticed in male FD patients compared to females (Table 3). The mean calculated vessel tortuosity score was 1.31 (range: 1.2–2.02) in males and 1.49 (range: 1.17–1.46) in females (*p* = 0.013). The subjective vessel tortuosity score presented significantly higher in males (mean = 2.58; range: 0.58–3) than females (mean = 1.08; range: 0.33–2.25; *p* = 0.005).

**Fig. 3:**
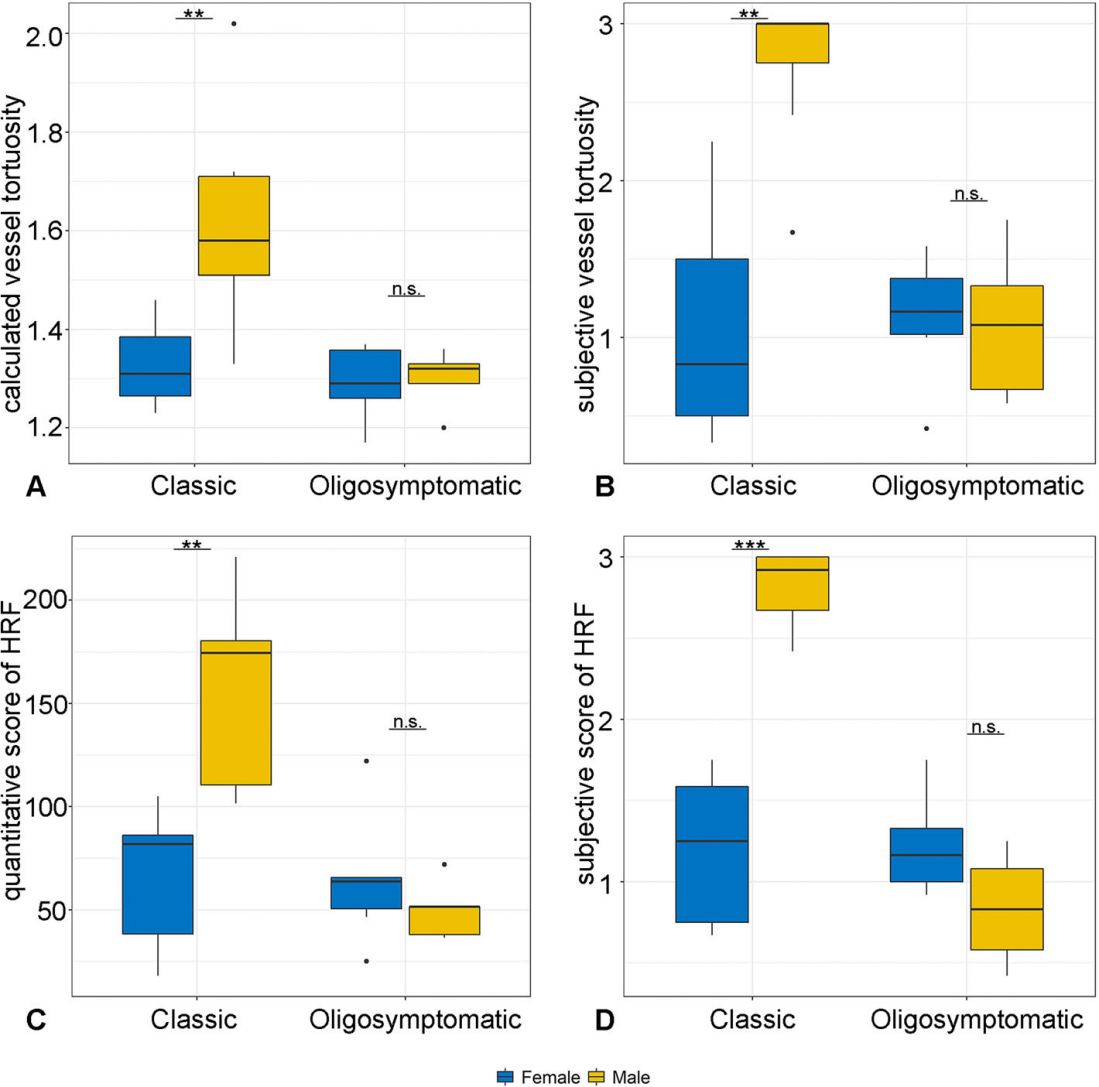
Subanalysis of the impact of the clinical course (classical or oligosymptomatic form) and gender of the FD cohort on calculated (**a**) and subjective (**b**) vessel tortuosity scores and quantitative (**c**) and subjective (**d**) scores of HRF. Male patients with the classic phenotype (*n* = 9) revealed significantly higher scores in calculated vessel tortuosity (*p* = 0.0081), subjective vessel tortuosity (*p* = 0.0018), quantitative score of HRF (*p* = 0.0015) and subjective score of HRT (*p* = 0.00098) in comparison with females with the classic phenotype (*n* = 7). No statistical difference was detected in the evaluated vessel tortuosity and HRF scores between the male (*n* = 5) and female FD patients (*n* = 6) with oligosymptomatic phenotype. Statistical analyses of data were performed with the Wilcoxon signed rank test. n.s.: not significant; *p < 0.05; **p < 0.01; ***p < 0.001

Further subanalyses were performed to characterize the impact of the clinical course (classical (*n* = 16 vs. oligosymptomatic form (*n* = 11)) and gender on mean quantitative and subjective score of HRF and vessel tortuosity. Classically affected patients (*n* = 16) presented with a significantly higher values in objective vessel tortuosity (*p* = 0.019), subjective vessel tortuosity (*p* = 0.033), quantitative score of HRF (*p* = 0.0096) and subjective score of HRT (*p* = 0.0066). In particularly male patients with the classic phenotype (*n* = 9) revealed significantly higher values in calculated vessel tortuosity (*p* = 0.0081), subjective vessel tortuosity (*p* = 0.0018), quantitative score of HRF (*p* = 0.0015) and subjective score of HRT (*p* = 0.00098) in comparison with females with the classic phenotype (*n* = 7). Patients with oligosymptomatic phenotype (*n* = 11) did not present gender specific score discrepancies in vessel tortuosity and HRT (Fig. 3).

The age of the investigated patients had no impact on the performed scores of HRF and vessel tortuosity.

Neither ERT nor the duration of the treatment did statistically affect the subjective and objective score of vessel tortuosity and HRT.

Furthermore, there was no significant correlation between the appearance or grade of CV and the quantitative and subjective score of HRF and the calculated and subjective vessel tortuosity score.

In addition, there was no significant difference in CRT and RNFL of the FD patients compared to the age-correlated control group (Table 2).

## Discussion

Beyond the classic ocular manifestations in FD, the presented study depicts novel intraretinal features in patients with FD outlined by SD-OCT imaging.

Intraretinal HRF have been observed in patients with retinitis pigmentosa and diabetic retinopathy, in which SD-OCT based HRF correlated with intraretinal accumulations and clinically obvious hard exudates [37–41]. To the best of our knowledge, SD-OCT-based intraretinal HRF have never been detected and discussed in patients with FD, as SD-OCT imaging is not routinely performed in patients with FD. HRF were detected predominantly within the inner retina (retinal nerve fiber layer - outer plexiform layer) sparing exclusively the outer nuclear layer and concomitantly photoreceptor integrity (Fig. 1).

In order to provide routine clinical feasibility, we graded the HRF on SD-OCT cross sections and achieved a strong correlation to the manually quantified amount of HRF. As automated or manual quantification of the HRF remains experimental or labor- and time-consuming, we suggest a simple SD-OCT based clinical grading system (grade 0–3; none, mild, moderate, severe) to estimate the burden of intraretinal HRF. Compared to an age-correlated control group of healthy volunteers a profound accumulation of HRF was detected on SD-OCT cross-sections in patients with FD.

Further longitudinal investigations are necessary to clarify whether the presented HRF are changing with disease progression or under treatment. Prospectively, an automated quantification of the HRF could be used for monitoring as such an automated segmentation of HRF in SD-OCT images of patients with diabetic retinopathy was reported recently [42].

Along the intraretinal cross-sectional analysis, we evaluated the previously described classical vascular tortuosity [30] in our cohort of patients. For that purpose, posterior pole cSLO images were selectively acquired to manually measure and clinically grade vessel tortuosity in the patient cohort as well as in the control group. As previously described, the analysis of the retinal vessels showed a significant increased vessel tortuosity in FD patients [30, 32, 33, 43–45]. On the basis of the previously published cumulative findings, our approach to estimate vessel tortuosity in our cohort was once more to simplify and increase clinical feasibility by a clinical grading system (grade 0–3; none, mild, moderate, severe). Manual quantification of the relative length (defined as the ratio between the true length of the considered vessel and the length of the underlying chord) as being previously performed by Sodi et al., encouraged the application of a clinical grading as both measurements correlated strongly [33]. Nevertheless as the field of ocular imaging by SD-OCT is rapidly evolving, future widely available quantification softwares might potentially overcome this subjective clinical grading.

Lyso-Gb3 is acknowledged as an important marker in FD and correlates with disease severity as well as ERT response [46–49]. Additionally, lyso-Gb3 contributes to identify high risk patients [46]. We therefore correlated the lyso-Gb3 levels of FD patients with the manually acquired vessel tortuosity score and the quantification of parafoveal HRF.

Interestingly, a strong correlation was present between the quantity of HRF and the concomitant lyso-Gb3. Quantification of vessel tortuosity also showed a moderate positive correlation with serum lyso-Gb3 levels. These findings align with the report by Sodi et al. who highlighted the correlation of vessel tortuosity and disease severity [30]. Furthermore, significantly higher score of HRF as well as higher vessel tortuosity scores were noticed in male FD patients compared to females.

A further subanalysis of the Fabry cohort revealed significantly higher HRF and vessel tortuosity scores in the classic phenotype of FD, in particularly in male individuals (Fig. 3), furthermore providing evidence of a causal relationship between the presence of HRF and disease severity.

## Conclusion

In summary, the present study describes novel intraretinal HRF in patients with FD and further provides a simple, yet differentiated grading system to estimate the presence of HRF as well as vessel tortuosity based upon noninvasive SD-OCT imaging. As FD is a multisystemic lysosomal storage disorder and clinical decision making is based on an interdisciplinary approach, we suggest SD-OCT imaging as a further adjunctive tool to increase the ophthalmologic risk assessment. Automated quantification of the intraretinal findings detected by SD-OCT imaging could serve as an easily accessible, quick monitoring tool. A major limitation of the present study is the cross-sectional design and the limited acquisition of global disease parameters. The underlying pathology remains to be explored as no histological and/or animal retinal documentations are published at present. As the retina and in particular the macular area are highly vascularized and perfused, capillary dysfunction and concomitant endothelial glycosphingolipids deposition [50] are potential explanations of the described HRF and may result in a pathologically hyperreflective capillary plexus of the inner retina.

Further investigations to clarify the retinal involvement have been initiated at our department. In addition, a prospective, longitudinal clinical study is currently ongoing at the university medical center Hamburg-Eppendorf that might further elucidate the impact of HRF in FD and the potential effect of systemic enzyme replacement therapy on the distribution of inner retinal HRFs.

## Data Availability

The datasets used and/or analysed during the current study are available from the corresponding author on reasonable request.

## Abbreviations

BCVA: Best-corrected visual acuity
CRT: Central retinal thickness
CV: Cornea verticillata
ESRD: End-stage renal disease
ETDRS: Early Treatment Diabetic Retinopathy Study
FD: Fabry disease
HRF: Hyperreflective foci
RNFL: Retinal nerve fiber layer
SD-OCT: Spectral domain optical coherence tomography
